# Jiawei Danxuan Koukang and Its Component Kaempferol Alleviate Oral Submucous Fibrosis by Restoring Epithelial‐Fibroblast Homeostasis and Suppressing Neutrophil Recruitment via the ANXA1/FPR2 Axis

**DOI:** 10.1002/fsn3.71785

**Published:** 2026-04-15

**Authors:** Yao Xiao, Yisi Tan, Yanli Liu, Ruiyi Chen, Tao Zhou, Zhaoyong Hu, Jin Tan

**Affiliations:** ^1^ Department of Stomatology The First Affiliated Hospital of Hunan University of Chinese Medicine Changsha China; ^2^ Medical School Hunan University of Chinese Medicine Changsha China; ^3^ Department of Health Management The First Affiliated Hospital of Hunan University of Chinese Medicine Changsha China

**Keywords:** ANXA1/FPR2, epithelial‐fibroblast homeostasis, jiawei danxuan koukang, kaempferol, neutrophil recruitment, oral submucous fibrosis

## Abstract

Oral submucous fibrosis (OSF) is a betel quid chewing‐associated precancerous disorder. Studies have shown that the traditional Chinese medicine Jiawei Danxuan Koukang (JDK) exhibits inhibitory effects on OSF, but its specific mechanisms and molecular targets remain unstated. This study aims to elucidate the pharmacological mechanisms of JDK and its component kaempferol in treating OSF. OSF rat model and in vitro cell models were induced by arecoline. Techniques, including hematoxylin–eosin (HE) staining, Masson staining, western blot, quantitative real‐time polymerase chain reaction (qRT‐PCR), enzyme‐linked immunosorbent assay (ELISA), immunofluorescence (IF) staining, co‐immunoprecipitation (Co‐IP), and molecular docking were used to evaluate the effects of JDK and kaempferol on OSF pathological damage, Annexin‐A1 (ANXA1) expression, neutrophil infiltration, collagen deposition, and fibroblast activation. JDK and kaempferol alleviated pathological damage in the oral mucosa tissues of OSF rats, inhibited collagen deposition and fibroblast activation marker expressions, including fibroblast activation protein (FAP), vimentin (VIM), alpha‐smooth muscle actin (α‐SMA), and matrix metalloproteinase‐1 (MMP1), and improved mouth opening function. Further studies revealed that JDK and kaempferol upregulated ANXA1 expression, thereby suppressing neutrophil recruitment, pro‐inflammatory cytokine release, and collagen deposition. In vitro experiments confirmed that kaempferol directly bound to ANXA1 in epithelial cells, enhancing its stability, and interacted with FPR2 signaling in fibroblasts to inhibit their activation, thereby restoring epithelial‐fibroblast homeostasis. However, knockdown of ANXA1 reversed these protective effects. JDK and kaempferol attenuated OSF by upregulating the ANXA1/FPR2 axis, inhibiting neutrophil infiltration and fibroblast activation, suggesting ANXA1 as a potential therapeutic target for OSF. To our knowledge, this is the first study to demonstrate that JDK/kaempferol exert anti‐fibrotic effects in OSF specifically through the ANXA1/FPR2 axis.

## Graphical abstract

JDK and kaempferol upregulate ANXA1 in oral epithelia, which bind to fibroblast FPR2 to suppress myofibroblast activation, and collagen deposition, while reducing neutrophil recruitment and inflammation, thereby restoring epithelial‐fibroblast homeostasis to alleviate arecoline‐induced OSF.

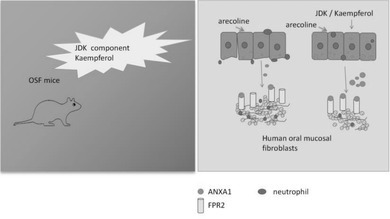

## 1 Introduction

Oral submucous fibrosis (OSF) is a common precancerous disease among betel nut chewers. It is characterized by chronic inflammation, progressive submucous fibrosis, and epithelial atrophy, leading to debilitating symptoms such as clenched teeth and an increased risk of oral malignant tumors (Chhabra et al. 2023; Jones et al. 2024). Although progress has been made in understanding its causes, the treatment strategies remain largely symptomatic rather than fundamental, focusing on symptom management rather than targeting the molecular drivers of fibrosis and malignant transformation (Jones et al. 2024). Therefore, it is of vital importance to clarify the pathogenic mechanism of OSF and search for therapeutic targets. Jiawei Danxuan Koukang (JDK), a modified formulation based on Tao Hong Si Wu Tang (THSWT) and Danxuan Koukang (DXKK), exhibits therapeutic potential in mitigating the progression of OSF. Studies have shown that JDK and its active ingredient quercetin inhibit arecoline‐induced OSF by suppressing the neurogenic locus notch homolog protein 1 (NOTCH1) pathway and the AR/eukaryotic translation initiation factor 5A2 (eIF5A2) pathway (Dai et al. 2025; Zhou et al. 2023). Moreover, JDK alleviates OSF by reducing inflammation, reshaping gut microbiota, and restoring metabolic homeostasis (C. Wang et al. 2026). Grounded in the traditional Chinese medicine principle of promoting blood circulation, removing blood stasis, detoxifying, and resolving hard lumps, the precise pharmacological pathways and target molecules through which JDK exerts its anti‐OSF effects in modern medical theory have yet to be fully elucidated. Analyzing the active ingredients and action pathways of JDK is of great significance for promoting the modernization of Chinese medicine and developing OSF‐specific drugs.

Studies have found that the potential mechanisms of OSF include the activation of fibroblasts, damage to the epithelial barrier, damage to endothelial cells, dysregulation of immune responses, and so on. Virtually all cellular components of the oral mucosa – encompassing epithelial cells, fibroblasts, vascular endothelial cells, and infiltrating immune cells – participate in and collectively contribute to the pathogenic mechanisms underlying OSF development (J. Tang et al. 2025). Recent studies have shown that arecoline induces epithelial cells to secrete miR‐17‐5p, which activates transforming growth factor‐β (TGF‐β) signaling in fibroblasts and promotes their differentiation into myofibroblasts that produce collagen (Xie et al. 2024). Furthermore, the spatial transcriptomic and metabolomic analyses of OSF‐derived oral squamous cell carcinoma (OSCC) highlighted the tumor microenvironment (TME) rich in ligand‐receptor networks, which promoted the pathological interactions among epithelial cells, fibroblasts, and immune cells (Zhi et al. 2024). Neutrophils, being the most abundant immune cell population, contribute substantially to key oncogenic processes including tumor initiation, progression, metastatic dissemination, and disease recurrence across multiple cancer types (Xiong et al. 2021). In conclusion, these findings emphasize the need for therapies that disrupt this intercellular communication to prevent fibrosis and canceration of the oral mucosa.

Annexin‐A1 (ANXA1), a glucocorticoid‐responsive protein, exhibits broad tissue distribution and plays a multifaceted role in oncogenesis through diverse molecular mechanisms (L. Li et al. 2024). In OSCC, reduced ANXA1 expression correlates with compromised epithelial integrity and exacerbated inflammatory processes (H. T. Wu, Chen, et al. 2021). Furthermore, overexpression of ANXA1 significantly reduced the proliferation, invasiveness and epithelial‐mesenchymal transition of OSCC cell lines, indicating the correlation of ANXA1 as a diagnostic/prognostic biomarker for OSCCs (Novizio et al. 2025; Wan et al. 2017). In addition to its role in inhibiting neutrophil chemotaxis and addressing cytokine storms, ANXA1 also maintains matrix balance by acting as a paracrine signaling molecule (Broering et al. 2024; X. Shen, Zhang, et al. 2020; You et al. 2024). In the research of laryngeal cancer, ANXA1/formyl peptide receptor 2 (FPR2) co‐localization exists in neutrophils, indicating that ANXA1 may be involved in the regulation of tumor growth and metastasis through the paracrine mechanism mediated by FPR2/lipoxin A4 receptor (ALX) (Gastardelo et al. 2014). Another study has shown that in the esophageal epithelium, ANXA1 binds to FPR2 on fibroblasts, preventing them from transforming into cancer‐associated fibroblasts (CAFs). However, in an environment lacking ANXA1, TGF‐β enhanced this process (Chen et al. 2023b). Thus, we hypothesize that ANXA1 deficiency accelerates OSF by driving fibroblast activation and neutrophil infiltration. It is worth noting that kaempferol has been proven to up‐regulate the expression of ANXA1 in the reproductive system, and molecular docking studies have confirmed that it has a strong binding affinity for ANXA1 (Zhao et al. 2021). Using high performance liquid chromatography–tandem mass spectrometry (HPLC/MS) analytical techniques, kaempferol was identified as a major bioactive component in JDK (Zhou et al. 2023). While JDK/kaempferol are known to be beneficial, their direct molecular target and the involvement of the ANXA1/FPR2 axis in OSF remain unexplored. This study aimed to clarify the pharmacological mechanism of JDK and its active component kaempferol, as well as the pathogenic mechanism of OSF. We proposed that JDK could treat OSF by up‐regulating ANXA1 to reverse epithelial fibrosis and inhibit neutrophil infiltration. Our research results might provide new therapeutic approaches for OSF, emphasizing that ANXA1 was a key target for preventing OSF and malignant transformation.

## 2 Materials and Methods

### 2.1 JDK Recipes and Sources

The JDK was provided by the First Affiliated Hospital of Hunan University of Chinese Medicine and consisted of: 10 g *Salvia miltiorrhiza Bunge*, 10 g *Scrophularia ningpoensis Hemsl*, 10 g *Angelica sinensis (Oliv.) Diels*, 5 g *Carthamus tinctorius Linn*, 10 g *Rehmannia henryi N. E. Brown*, 10 g *Hedyotis diffusa Willd*, 10 g *Astragalus saxorum Simps*, 10 g *Mentha haplocalyx Briq*, 10 g *Platycodon grandiflorus (Jacq.) A. DC*, 10 g *Prunella vulgaris Linn*, 10 g *Artemisia capillaris Thunb*, *and* 10 g *Paeonia lactiflora Pall*. The qualitative and quantitative analysis of compounds in JDK has been presented in our previous studies (Zhou et al. 2023).

### 2.2 Animal Modeling and Intervention

Adult male Sprague–Dawley rats (SPF grade, 7–8 weeks old, weighing 180–200 g) were obtained from Hunan SJA Laboratory Animal Co. Ltd. (Changsha, China). The rats were housed in a sterile environment maintained at 22°C–28°C with a 12 h light/dark cycle. Prior to experimentation, all rats were allowed to adapt to the housing conditions for 7 days with free access to food and water. Rats were randomly divided into 6 groups: Sham, OSF, OSF + JDK, OSF + Kaempferol, OSF + Kaempferol + sh‐NC, and OSF + Kaempferol + sh‐ANXA1, with 6 rats in each group. In the OSF group, after anesthetizing the rats with sodium pentobarbital (50 mg/kg) (N. Li et al. 2018), 0.2 mL of arecoline (10 mg/mL, dissolved in 0.9% normal saline, S25609‐25 g, Shanghai yuanye Bio‐Technology Co. Ltd., China) was injected into the bilateral buccal mucosa (Chiang et al. 2020). Following drug administration, the rats were fasted (withheld from food and water) for 2 h. Then, a brush was used to dip arecoline solution and brush the buccal mucosa on both sides of the rats 20 times each. The above operation should be carried out once a day for 8 weeks. Rats in the Sham group were injected with the same amount of normal saline. After the successful modeling, intervention treatment was initiated for rats in each group. Rats in the OSF + JDK group were gavaged with JDK (12 g/kg, five days a week) (Zhou et al. 2023). Rats in the OSF + Kaempferol group were gavaged with kaempferol (50 mg/kg/d, S25632, Shanghai yuanye Bio‐Technology Co. Ltd., China) (M. Wang et al. 2024). Rats in OSF + Kaempferol+sh‐NC and OSF + Kaempferol+sh‐ANXA1 groups were respectively injected with 20 μL of sh‐NC/sh‐ANXA1 lentivirus (1 × 10^9^ TU/mL, 7 days) (GAGATTTTGACAACAAGATCT, HG‐LV012904sh0475, HonorGene, China) under the buccal mucosa before intragastric administration of kaempferol (Du et al. 2019). After 7 weeks of treatment, the rats were euthanized by intraperitoneal injection of sodium pentobarbital (150 mg/kg) (Y. Li et al. 2022). Blood and oral mucosal tissues were collected for further analysis. This study was approved by the Laboratory Animal Ethics Committee of Hunan University of Chinese Medicine (HN‐LL‐GZR‐2023‐43).

### 2.3 Measurement of the Maximal Mouth Opening of Rats

After anesthetizing the rats, the mouths were opened with a force gauge (F = 2 N), and the margins of the upper and lower central incisors of the rats before administration, 2 weeks after administration, and 7 weeks after administration were measured respectively with a vernier caliper. Measurements were taken three times and averaged to 0.01 mm precision (Xuan et al. 2024).

### 2.4 Cell Culture

The human oral mucosal epithelial cell line (HUM‐iCell‐m004, iCell, China) was maintained in specialized epithelial cell culture medium (AW‐MC019, Abiowell, China). For human oral mucosal fibroblasts (HTX2904, ScienCell, USA), Dulbecco's modified Eagle's medium (DMEM, D5796, Sigma, USA) was used as the growth medium. Both culture media were supplemented with 10% fetal bovine serum (FBS, 10099141, Gibco, USA) and 1% Penicillin/Streptomycin solution (SV30010, Beyotime, China). Cell cultivation was performed in a humidified CO2 incubator (DH‐160I, SANTN, China) under standard conditions (37°C, 5% CO2). Cell line authentication was confirmed through short tandem repeat (STR) analysis.

### 2.5 Cell Grouping and Treatment

To screen the appropriate arecoline concentration for constructing the in vitro model of OSF, human oral mucosal epithelial cells were treated with arecoline at 0, 0.05, 0.1, 0.15, 0.2, and 0.3 mM for 24 h respectively (Zhou et al. 2023). Similarly, to screen for the appropriate concentration of kaempferol for intervention, human oral mucosal epithelial cells were treated with kaempferol at concentrations of 0, 1.25, 2.5, 5, 10, 20, and 40 μM for 24 h respectively. Cell viability was then assessed using the cell counting kit‐8 (CCK‐8) method. To screen for the appropriate knockdown site of ANXA1, human oral mucosal epithelial cells were transfected with sh‐NC (TTCCCGAGTTAACCAATTTAC), sh‐ANXA1#1 (GACATTTATGAGATAAAGGAA), sh‐ANXA1#2 (GCCAAAAGAATGTCTGTTTCT), and sh‐ANXA1#3 (GGCTAAGACTTGGCTTCATTT) plasmids, respectively, using Lipofectamine 2000 (11,668,019, Invitrogen, USA). In subsequent experiments, human oral mucosal epithelial cells were divided into Control, OSF, OSF + Kaempferol, OSF + Kaempferol + sh‐NC, and OSF + Kaempferol + sh‐ANXA1 groups. Control group cells were maintained under standard culture conditions. The cells in the OSF group were treated with 0.2 mM arecoline for 24 h. The cells in the OSF + Kaempferol group were treated with 0.20 mM arecoline and 20 μM kaempferol for 24 h. The cells in OSF + Kaempferol+sh‐NC/OSF + sh‐NC/OSF + Kaempferol + sh‐ANXA1 groups were first transfected with the sh‐NC/sh‐ANXA1 plasmids (HG‐SH145868, HonorGene, China), and then treated with 0.20 mM arecoline and 20 μM kaempferol for 24 h.

To explore the crosstalk between epithelial cells and fibroblasts in the oral mucosa, a non‐contact Transwell co‐culture system (0.4 μm, 3412, Corning, USA) was employed. Untreated oral mucosal fibroblasts were seeded in the lower chamber of a 6‐well plate. Oral mucosal epithelial cells, subjected to the various treatments listed below, were seeded in the upper chamber. The cells were co‐cultured for 24 h at a 1:1 seeding ratio to simulate paracrine communication. The treatment groups for epithelial cells included OSF, OSF + Kaempferol, OSF + rANXA1, OSF + Kaempferol+Boc1, OSF + Kaempferol+sh‐NC, and OSF + Kaempferol+sh‐ANXA1. Among them, the epithelial cells in the OSF + rANXA1 group were treated with 0.20 mM arecoline and 100 nM recombinant ANXA1 (rANXA1) for 24 h (Barbosa et al. 2019). The epithelial cells of the OSF + Kaempferol+Boc1 group were treated with 0.20 mM arecoline, 20 μM kaempferol, and 10 μM Boc1 (FPRs antagonist) for 24 h (Liu et al. 2016). In addition, fibroblasts transfected with sh‐NC (TTCCGAGTTAACCAATTTAC) or sh‐FPR2 plasmids (GAGATAAGAACCAATATGGAT, HG‐SH001462, HonorGene, China) were co‐cultured with epithelial cells from the OSF + Kaempferol group for 24 h.

### 2.6 Hematoxylin–Eosin (HE) Staining

Oral mucosal tissue samples were fixed with 4% paraformaldehyde (AWI0056a, Abiowell, China), embedded in paraffin (10,023,418, Shanghai Sinopharm Chemical Reagent Co. Ltd., China), and sectioned for HE staining to assess pathological changes. Slices were dewaxed in xylene for 20 min, then dehydrated using a graded ethanol series (100%–75%), 5 min per concentration. They were stained with hematoxylin (AWI0001a, Abiowell, China) for 1 min, rinsed with distilled water, and blued in phosphate‐buffered saline (PBS, AWI0129a, Abiowell, China). Afterwards, slices were stained with eosin (AWI0029a, Abiowell, China) for 1 min, rinsed with distilled water, and dehydrated again with a graded alcohol series (95%–100%), 5 min per concentration. Finally, slices were cleared in xylene for 10 min, mounted with neutral gum (AWI0238a, Abiowell, China), and examined under a microscope (BA210T, Motic, China).

### 2.7 Masson Staining

Masson staining was conducted to evaluate collagen deposition in oral mucosal tissues. Slides were dewaxed and hydrated. They were stained with hematoxylin solution for 1 min, rinsed sequentially with tap water and distilled water, and then soaked in PBS (pH 7.4) for 10 min to blue the nuclei. Next, slides were stained with acid fuchsin for 5 min, followed by a 30‐s treatment with phosphomolybdic acid differentiation solution. The tissues were counterstained with aniline blue for 3 min and rinsed with absolute ethanol. After air‐drying, slides were cleared in xylene and mounted with neutral gum for microscopic examination.

### 2.8 Immunohistochemistry (IHC) Staining

After dewaxing and hydration, oral mucosal tissue slices were immersed in citrate buffer (0.01 M, pH 6.0) (AWI0206a, Abiowell, China) and boiled for antigen retrieval. Endogenous enzymes were inactivated with 1% periodic acid (AWI0528a, Abiowell, China). Slices were then incubated overnight with the primary antibody for ANXA1 (1:200, 55,018–1‐AP, Proteintech, USA), followed by poly‐HRP‐anti‐mouse‐IgG. Diaminobenzidine was added for color development. The slices were counterstained with hematoxylin, rinsed with distilled water, blued in PBS, and dehydrated using a graded alcohol series (60%–100%). After clearing in xylene and mounting with neutral gum, the sections were examined under a microscope.

### 2.9 Immunofluorescence (IF) Staining

Cell slides were washed with PBS, fixed with 4% paraformaldehyde, permeabilized with 0.3% Triton X‐100, and blocked with 5% bovine serum albumin (BSA). For tissue slices, antigen retrieval was performed after dewaxing to water. The tissue slices were washed with PBS, treated sequentially with sodium borohydride, 75% ethanol, and Sudan black dye, followed by another PBS wash and blocking with 5% BSA. The cell slides were incubated respectively with primary antibodies of ANXA1 (1:100, ab268070, Abcam, UK), FPR2 (1:200, ab63023, Abcam, UK), and alpha‐smooth muscle actin (α‐SMA, 1:50, BM0002, Boster, USA). The tissue slices were incubated respectively with primary antibodies of Ly6G (1:200, AWA55855, Abiowell, China). The cell slides and tissue slices were incubated with CoraLite488‐conjugated Goat Anti‐Rabbit IgG (H + L) (1:200, SA00013‐2, Proteintech, Chicago, IL, USA) or CoraLite594‐conjugated Goat Anti‐Mouse IgG (H + L) (1:200, SA00013‐3, Proteintech, Chicago, IL, USA). After washing, nuclei were stained with 4′,6‐diamidino‐2‐phenylindole (DAPI, AWI0331a, Abiowell, China) and rinsed with PBS. Samples were mounted with buffered glycerin and imaged using a microscope (BA410T, Motic, Germany).

### 2.10 Quantitative Real‐Time Polymerase Chain Reaction (qRT‐PCR)

Total RNA was isolated from oral mucosal tissues and epithelial cells with Trizol reagent (15,596,026, Thermo, USA). cDNA synthesis was carried out using an mRNA reverse transcription kit (CW2569, CWBIO, China). Quantitative real‐time PCR (qRT‐PCR) was conducted on an ABI QuantStudio1 Real‐Time PCR System with UltraSYBR Mixture (CW2601, CWBIO, China). Gene expression levels were calculated using the 2^−ΔΔCt^ method, normalized to β‐actin as the internal control. Primer sequences used in the study are listed in Table 1.

**TABLE 1:** Primer sequences.

Gene	Sequence	Length (bp)
H‐β‐Actin	Forward: ACCCTGAAGTACCCCATCGAG	224
	Reverse: AGCACAGCCTGGATAGCAAC	
H‐ANXA1	Forward: ACAGATCAAAGCAGCATATCTCC	142
	Reverse: AGCACGAAGTTCATCAGCATC	
R‐β‐Actin	Forward: ACATCCGTAAAGACCTCTATGCC	223
	Reverse: TACTCCTGCTTGCTGATCCAC	
R‐ANXA1	Forward: GCGTTCTAGCTGTTTGCGAG	125
	Reverse: GCCACACCTAGCAACCAAAG	

### 2.11 Western Blot

Protein extraction from oral mucosal tissues and epithelial cells was performed using RIPA lysis buffer (AWB0136, Abiowell, China). The isolated proteins were then separated via SDS‐PAGE and subsequently transferred onto nitrocellulose (NC) membranes. To prevent nonspecific binding, the membranes were blocked with 5% skim milk (AWB0004, Abiowell, China) for 1.5 h. Next, they were incubated overnight at 4°C with primary antibodies and later with secondary antibodies at room temperature for another 1.5 h. Chemiluminescent signals were developed using Super ECL Plus detection reagent (AWB0005, Abiowell, China). Band intensities were quantified using Quantity One software (ver. 4.6.6, Bio‐Rad Inc., USA), with β‐actin serving as the loading control. Detailed antibody information is provided in Table 2.

**TABLE 2:** The information about antibodies.

Name	Dilution rate	Cat number	Source	Company	Country
FAP	1: 1000	15,384–1‐AP	Rabbit	Proteintech	USA
VIM	1: 5000	10,366–1‐AP	Rabbit	Proteintech	USA
α‐SMA	1: 2000	55,135–1‐AP	Rabbit	Proteintech	USA
MMP1	1: 1000	ab137332	Rabbit	Abcam	UK
ANXA1	1: 25000	21,990–1‐AP	Rabbit	Proteintech	USA
ZO‐1	1: 5000	21,773–1‐AP	Rabbit	Proteintech	USA
Occludin	1: 8000	13,409–1‐AP	Rabbit	Proteintech	USA
FPR2	1: 6000	ab63023	Rabbit	Abcam	UK
β‐Actin	1: 5000	66,009–1‐Ig	Mouse	Proteintech	USA
HRP goat anti‐mouse IgG	1: 5000	SA00001‐1	Mouse	Proteintech	USA
HRP goat anti‐rabbit IgG	1: 6000	SA00001‐2	Rabbit	Proteintech	USA

### 2.12 Enzyme‐Linked Immunosorbent Assay (ELISA)

The supernatant was collected from pretreated whole blood and cell cultures for subsequent analysis. Interleukin‐6 (IL‐6, CSB‐E04638h, CUSABIO, China), IL‐1β (KE00021, Proteintech, USA), and tumor necrosis factor‐alpha (TNF‐α, KE00154, Proteintech, USA) ELISA kits were utilized in cell supernatant, while IL‐6 (KE20024, Proteintech, USA), IL‐1β (KE00021, Proteintech, USA), TNF‐α (KE20018, Proteintech, USA), and ANAX1 (ER2038, Wuhan Fine Biotech Co. Ltd., China) ELISA kits were utilized in serum.

### 2.13 CCK‐8

Human oral mucosal epithelial cells in the logarithmic growth phase were detached using trypsin (AWC0232, Abiowell, China). The cells were seeded into 96‐well plates at a density of 1 × 10^4^ cells per well, with 100 μL of medium in each well. Following cell attachment, the cultures were exposed to designated drug treatments for 24 and 48 h. Subsequently, 10 μL of CCK‐8 solution (NU679, Dojindo, Japan) was introduced into each well, followed by incubation at 37°C for 4 h. Absorbance at 450 nm was measured using a microplate reader (MB‐530, HEALES, China).

### 2.14 Drug Affinity Responsive Target Stability (DARTS) Assay

Human oral mucosal epithelial cells (1 × 10^7^) were lysed in RIPA lysis buffer, in which phosphatase and protease inhibitors were added. After collecting the cell lysate, the samples were equally divided into 5 portions and co‐incubated with different concentrations of kaempferol (5, 10, and 20 μM) at room temperature for 2 h. Subsequently, the lysates were digested with proteinase K (at a mass ratio of 1:400) at 37°C for 30 min. Finally, the expression level of ANXA1 protein was detected by western blot (Wu, Li, et al. 2021).

### 2.15 Cellular Thermal Shift Assay (CETSA)

Human oral mucosal epithelial cells were lysed using a freeze–thaw method with liquid nitrogen. The cell lysates were then divided into two aliquots: one was incubated with DMSO as a control group, and the other was incubated with 20 μM kaempferol for 30 min. Subsequently, the lysates from both groups were heated at gradually increasing temperatures (39°C–63°C, in 4°C increments) for 5 min each. After boiling for 10 min, western blot analysis was performed to determine the abundance level of ANXA1 (Wu, Li, et al. 2021).

### 2.16 Flow Cytometry

Human oral mucosal epithelial cells digested with EDTA‐free trypsin were collected and washed twice in PBS (2000 rpm, 5 min) before being resuspended for further use. Subsequently, 500 μL of Binding Buffer was added to the cells for resuspension. Annexin V‐FITC and Propidium Iodide (5 μL each, KGA1030, KeyGEN BioTECH, China) were sequentially added to the cell suspension. The apoptosis rates of cells were measured with a flow cytometer (A00‐1‐1102, Beckman, USA) following a 10‐min dark incubation at room temperature.

### 2.17 Co‐Immunoprecipitation (Co‐IP)

Proteins were isolated from cells using RIPA lysis buffer, followed by overnight incubation at 4°C with specific antibodies: ANXA1 (21990–1‐AP, Proteintech, USA), FPR1 (ab113531, Abcam, UK), FPR2 (ab63023, Abcam, UK), FPR3 (ab172908, Abcam, UK), or rabbit IgG (B900610, Proteintech, USA). Subsequently, 10 μL of protein A/G agarose beads were added to the mixture and incubated at 4°C for 2 h. The agarose beads with immune complexes were washed three times with lysis buffer, centrifuged at 3000 rpm for 3 min, and then heated in sample buffer for 5 min. After centrifugation, the supernatant was collected for western blot analysis.

### 2.18 Molecular Docking Verification of Kaempferol and ANXA1

The three‐dimensional (3D) structure diagram of ANXA1 was retrieved from the Protein Data Bank (PDB) database (https://www.rcsb.org/), and processed using PyMOL (ver. 2.3.1) to eliminate water molecules. Subsequently, the structure was loaded into Autodock tools (ver. 1.5.6) for hydrogen atom addition, charge calculation, and atom type assignment before being exported in PDBQT format. The Chemical Abstracts Service (CAS) number of kaempferol was obtained from the PubChem database (https://pubchem.ncbi.nlm.nih.gov/), and its 3D structure diagram was downloaded. Energy minimization was performed using Chem3D Pro, and the optimized structure was saved in mol2 format. Molecular docking was carried out with VINA software (ver. 1.1.2, developed by Dr. Oleg Trott), and the docking results were visualized using Discovery Studio. The final 3D molecular docking model was generated and exported.

### 2.19 Statistical Analysis

The statistical analysis was performed using GraphPad Prism software (ver. 8.0.1, GraphPad Software Inc., USA), with results presented as mean ± SD. Normality and homogeneity of variance were assessed using the Kolmogorov–Smirnov test along with descriptive statistical analysis. For intergroup comparisons, one‐way ANOVA followed by Tukey's multiple comparison test was applied. Two‐way ANOVA with Bonferroni correction was utilized to examine differences across various time points and temperature conditions. Pearson correlation analysis was conducted to evaluate the relationships between ANXA1, Ly6G, FAP levels, and maximal mouth opening. A *p*‐value less than 0.05 was considered statistically significant. To minimize bias, all experiments incorporated randomization and blinded evaluation procedures.

## 3 Results

### 3.1 JDK And Kaempferol Alleviated the Pathological Damage of Arecoline‐Induced OSF Rats

**FIGURE 1:**
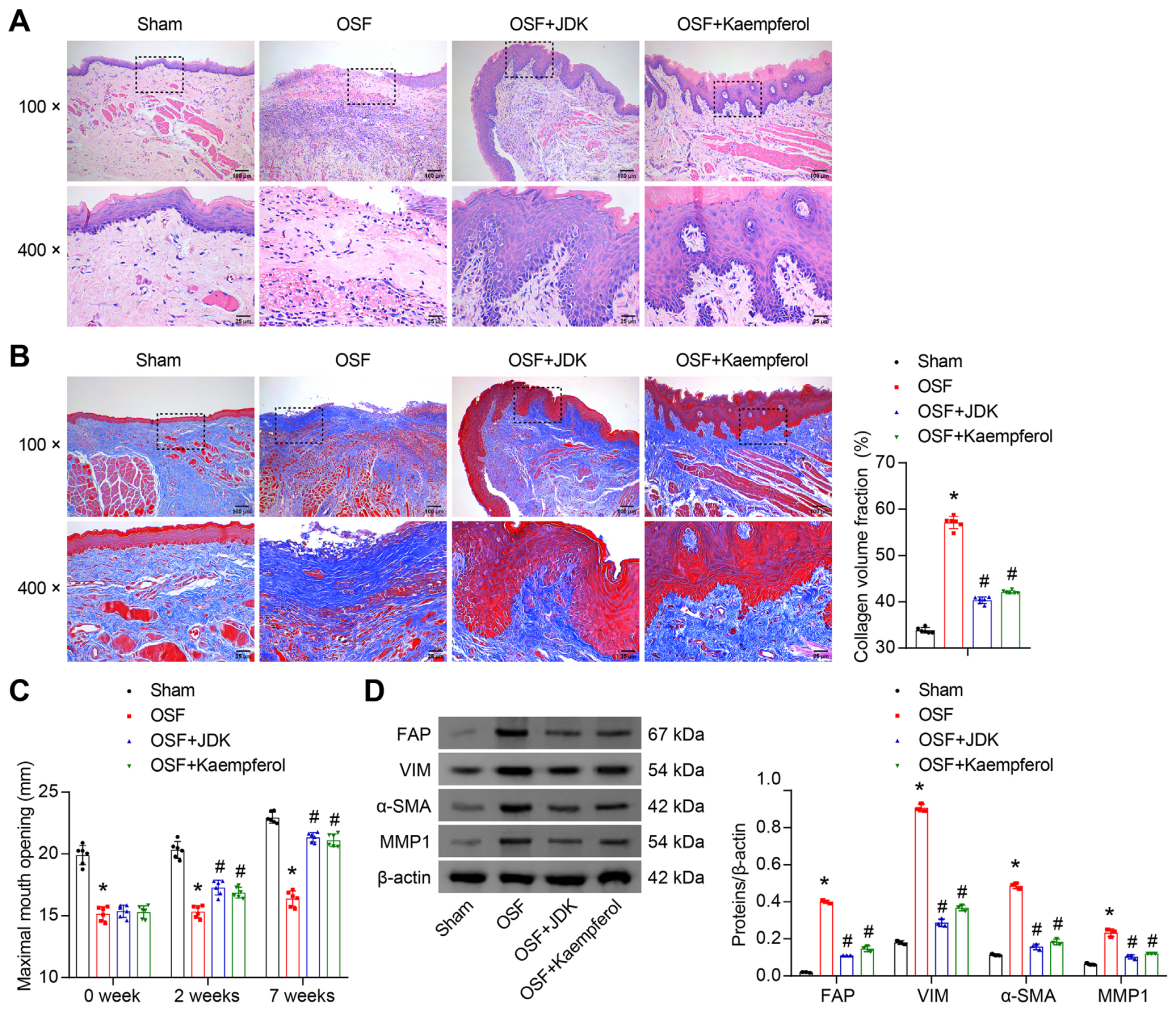
JDK and kaempferol ameliorated arecoline‐induced pathological damage in OSF rats. (A) HE staining analysis of morphological changes in oral mucosal tissues (100×, scale bar = 100 μm; 400×, scale bar = 25 μm). (B) Masson staining analysis of collagen fiber deposition in the oral submucosa (100×, scale bar = 100 μm; 400×, scale bar = 25 μm). (C) Maximal mouth opening at different treatment time points post‐modeling. (D) Western blot analysis of fibroblast activation markers (FAP, VIM, α‐SMA, and MMP1) in oral mucosal tissues. Data are presented as mean ± SD (*n* = 6). *p < 0.05 vs. Sham, ^#^ *p* < 0.05 vs. OSF.

To evaluate the in vivo therapeutic effects of JDK and its component kaempferol on OSF, we established an OSF rat model by intraoral mucosal injection of arecoline and administered JDK or kaempferol via gavage for 7 weeks. HE staining revealed that in the Sham group, the keratinized layer of the rat oral mucosa appeared light pink and uniform, with a clear epithelial boundary and no inflammatory cell infiltration. In contrast, the OSF group exhibited oral mucosal epithelial atrophy, disorganized structure, muscle tissue atrophy replaced by dense fibrous tissue, reduced submucosal vasculature, and diffuse inflammatory cell infiltration. However, in the OSF + JDK and OSF + Kaempferol groups, the oral mucosal epithelial structure was largely restored, and no inflammatory infiltration was observed (Figure 1A). Masson staining further demonstrated that compared to the Sham group, the OSF group showed extensive collagen fiber deposition in the oral submucosa. In contrast, the OSF + JDK and OSF + Kaempferol groups exhibited reduced collagen deposition (Figure 1B). To mimic the restricted mouth opening observed in human OSF patients, we measured the maximal mouth opening of rats at different treatment time points post‐modeling. The results displayed that the OSF group had a significantly decreased maximum mouth opening compared to the Sham group, indicating fibrosis‐induced tissue stiffness and functional impairment. However, after treatment with JDK or kaempferol, the maximal mouth opening increased, suggesting attenuated fibrosis in a time‐dependent manner (Figure 1C). Furthermore, western blot analysis of fibroblast activation markers in oral mucosal tissues revealed that compared to the Sham group, the OSF group exhibited upregulated expression of FAP, VIM, α‐SMA, and MMP1. However, treatment with JDK or kaempferol significantly downregulated the expression of these proteins (Figure 1D). These findings indicated that JDK and kaempferol effectively alleviated arecoline‐induced oral mucosal damage, inflammation, and fibrosis by inhibiting collagen deposition and suppressing profibrotic protein expression, thereby improving mouth opening function.

### 3.2 JDK And Kaempferol Upregulated ANXA1 Expression in the Oral Mucosa of OSF Rats

**FIGURE 2:**
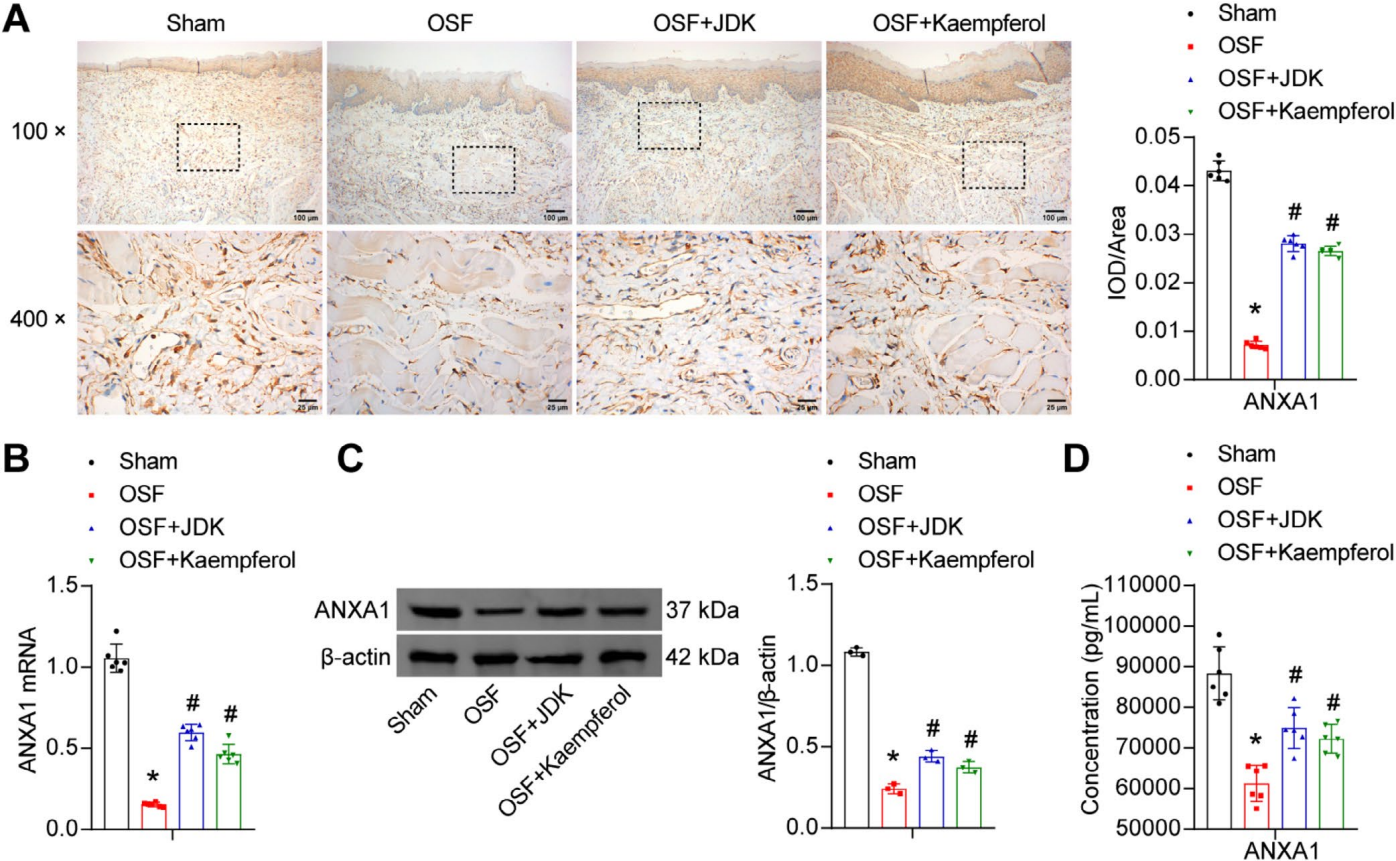
JDK and kaempferol promoted ANXA1 expression in the oral mucosa of OSF rats. (A) IHC staining analysis of ANXA1 distribution in oral mucosal tissues (100×, scale bar = 100 μm; 400×, scale bar = 25 μm). (B) qRT‐PCR analysis of ANXA1 mRNA expression in oral mucosal tissues. (C) Western blot analysis of ANXA1 protein expression in oral mucosal tissues. (D) ELISA measurement of ANXA1 concentration in serum. Data are presented as mean ± SD (*n* = 6). *p < 0.05 vs. Sham, ^#^ *p* < 0.05 vs. OSF.

Previous experiments confirmed the anti‐inflammatory and anti‐fibrotic effects of JDK and kaempferol, but their molecular targets remained unclear. We thus focused on ANXA1, an endogenous regulatory protein with both anti‐inflammatory and anti‐fibrotic potential. Studies have shown that ANXA1 exerts anti‐inflammatory effects by suppressing neutrophil recruitment and inhibiting pro‐inflammatory mediator production (Ni et al. 2021; Sugimoto et al. 2016). Research on epithelial‐fibroblast crosstalk via the ANXA1‐FPR2 axis has demonstrated that loss of ANXA1 function in premalignant or malignant epithelial cells can activate normal fibroblasts, promoting their transformation into cancer‐associated fibroblasts (CAFs) (Chen et al. 2023b). To determine whether ANXA1 mediates the anti‐inflammatory and anti‐fibrotic effects of JDK and kaempferol, we measured ANXA1 levels in rat oral mucosal tissues and serum. The results showed that arecoline treatment downregulated ANXA1 expression at both the gene and protein levels in epithelial cells and reduced ANXA1 secretion into the bloodstream. However, JDK or kaempferol intervention partially restored ANXA1 synthesis and secretion (Figure 2). These findings suggested that JDK and kaempferol upregulated ANXA1 expression, thereby exerting anti‐inflammatory and anti‐fibrotic effects.

### 3.3 JDK And Kaempferol Suppressed Neutrophil Recruitment and Collagen Deposition in OSF Rats by Upregulating ANXA1 Expression

**FIGURE 3:**
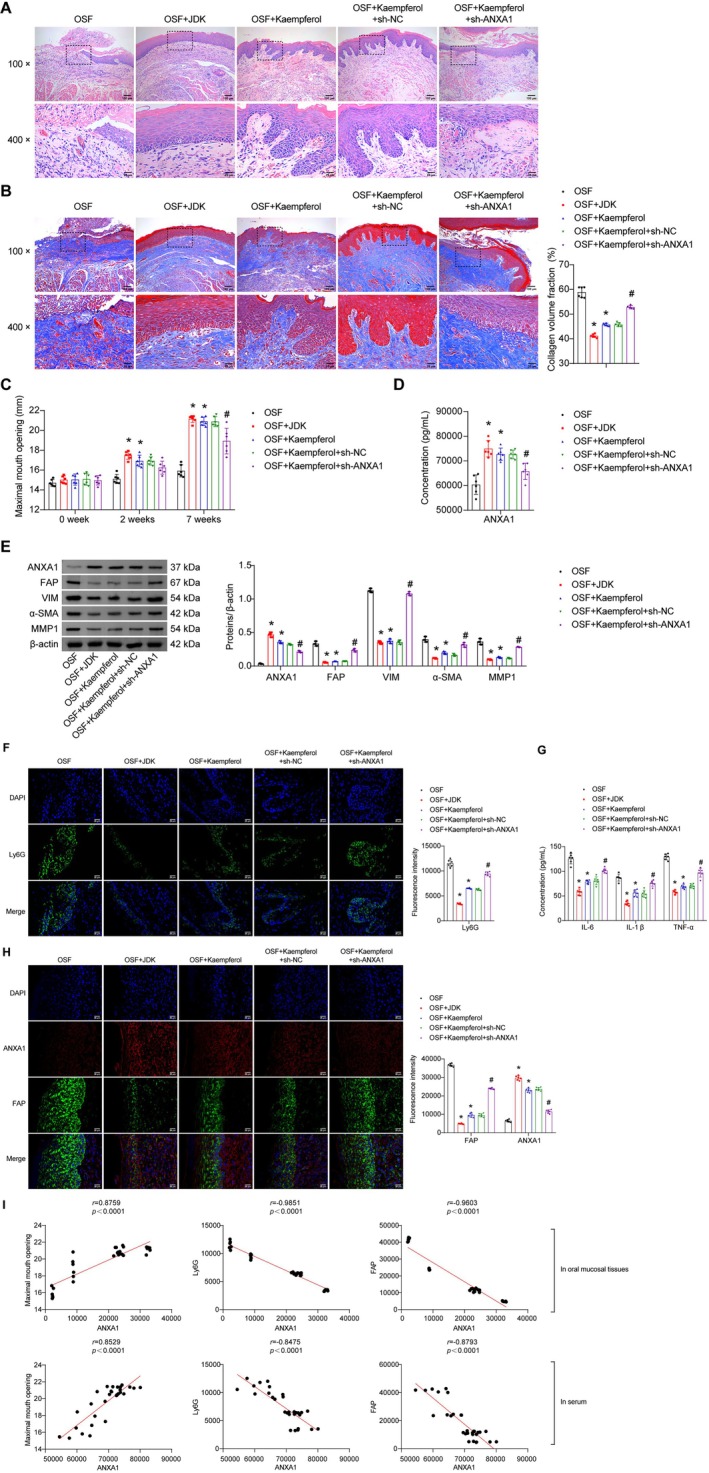
ANXA1 knockdown reversed the inhibitory effects of JDK and kaempferol on neutrophil recruitment and collagen deposition in OSF rat oral mucosa. (A) HE staining analysis of morphological changes in oral mucosal tissues (100×, scale bar = 100 μm; 400×, scale bar = 25 μm). (B) Masson staining analysis of collagen fiber deposition in oral submucosa (100×, scale bar = 100 μm; 400×, scale bar = 25 μm). (C) Maximal mouth opening at different treatment time points post‐modeling. (D) ELISA analysis of ANXA1 concentration in serum. (E) Western blot analysis of fibroblast activation markers (FAP, VIM, α‐SMA, and MMP1) in oral mucosal tissues. (F) IF staining of Ly6G expression in oral mucosa (scale bar = 25 μm). (G) ELISA measurement of IL‐6, IL‐1β, and TNF‐α concentrations in serum. (H) IF co‐staining of ANXA1 and FAP in oral mucosa (scale bar = 25 μm). (I) Pearson correlation analysis between ANXA1 expression (in tissues and serum) and Ly6G, FAP levels, or maximal mouth opening. Data are presented as mean ± SD (*n* = 6). *p < 0.05 vs. OSF, ^#^ *p* < 0.05 vs. OSF + Kaempferol + sh‐NC.

Given the previous findings demonstrating ANXA1‐mediated anti‐inflammatory and anti‐fibrotic effects of JDK and kaempferol in OSF treatment, we conducted further in vivo validation through lentivirus‐mediated ANXA1 knockdown in rats. Histomorphological analysis revealed that JDK and kaempferol ameliorated arecoline‐induced oral mucosal lesions and collagen fiber deposition. However, ANXA1 knockdown reversed these therapeutic effects (Figure 3A,B). Further analysis demonstrated that the maximal mouth opening of OSF rats gradually recovered over time following JDK and kaempferol intervention. In contrast, ANXA1 knockdown exacerbated oral fibrosis, leading to reduced maximal mouth opening in OSF rats (Figure 3C). Western blot results showed that the promotive effects of JDK and kaempferol on ANXA1 synthesis and serum secretion were inhibited by ANXA1 knockdown. Moreover, the suppressive effects of JDK and kaempferol on fibroblast activation markers (FAP, VIM, α‐SMA, and MMP1) were reversed after ANXA1 knockdown (Figure 3D,E). To investigate the role of ANXA1 deficiency in neutrophils, we performed IF staining of the oral mucosa using Ly6G as a neutrophil marker. Compared with the Sham group, OSF rats exhibited significantly elevated Ly6G expression in oral mucosa, indicating substantial neutrophil recruitment. JDK or kaempferol treatment effectively reduced Ly6G expression, suggesting attenuated neutrophil migration. However, ANXA1 knockdown partially counteracted the inhibitory effects of JDK or kaempferol on neutrophil recruitment (Figure 3F). Subsequent ELISA analysis of serum inflammatory factors (IL‐1β, IL‐6, TNF‐α) revealed that JDK or kaempferol treatment significantly suppressed arecoline‐induced inflammatory cytokine release. However, ANXA1 knockdown partially reversed this suppression (Figure 3G). Additional IF staining demonstrated that JDK or kaempferol intervention promoted ANXA1 secretion and inhibited FAP expression in OSF rat oral mucosa, while ANXA1 knockdown altered these molecular changes (Figure 3H). Pearson correlation analysis showed negative correlations between ANXA1 expression and both Ly6G and FAP levels in oral mucosal tissues and serum. Furthermore, the maximal mouth opening was positively correlated with ANXA1 expression in both oral mucosa and serum (Figure 3I). These above results revealed that upregulation of ANXA1 expression mediated the inhibitory effects of JDK and kaempferol on neutrophil recruitment and collagen deposition in OSF rat oral mucosa.

### 3.4 Kaempferol Alleviated Arecoline‐Induced Cell Damage by Up‐Regulating the Expression of ANXA1 in the in Vitro OSF Model

**FIGURE 4:**
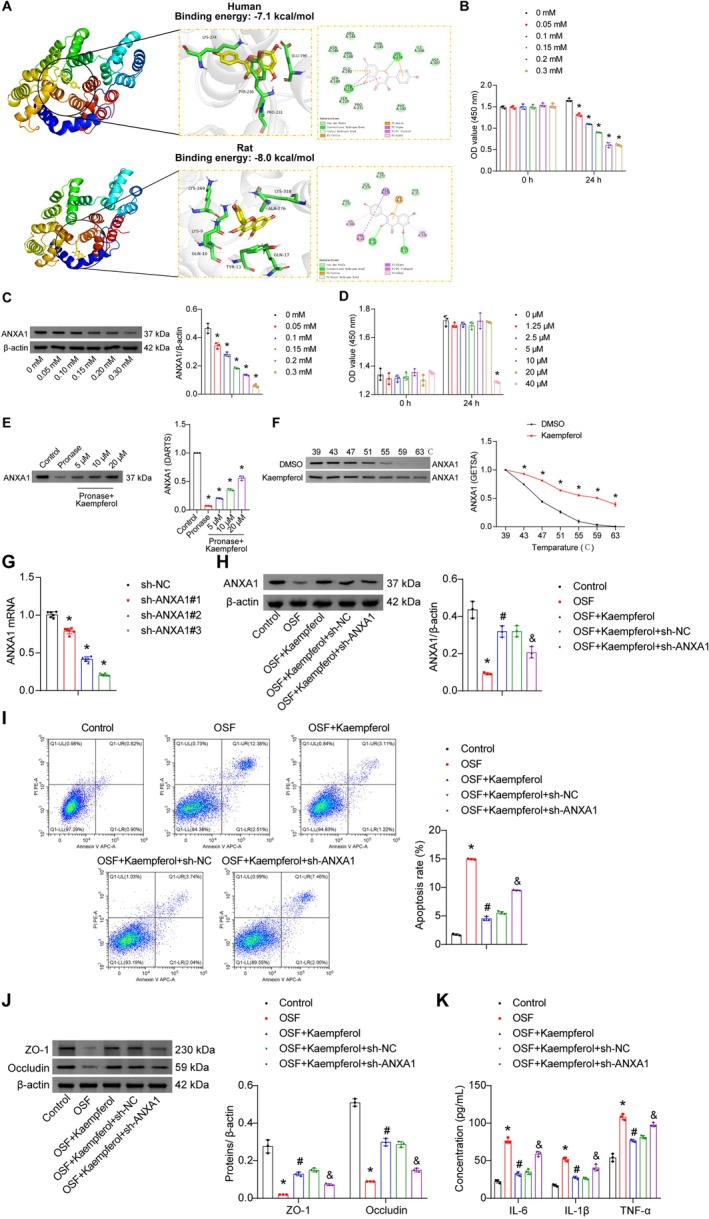
Kaempferol alleviated arecoline‐induced oral mucosal epithelial cell injury by binding and upregulating ANXA1. (A) Molecular docking conformations of kaempferol with human and rat ANXA1. (B) Cell viability assessed by CCK‐8 assay after 24 h treatment with various arecoline concentrations (0–0.30 mM). * *p* < 0.05 vs. 0 mM. (C) Western blot analysis of ANXA1 protein expression. (D) Cell viability assessed by CCK‐8 assay after 24 h treatment with various kaempferol concentrations (0–40 μM). *p < 0.05 vs. 0 μM. (E) Western blot analysis of ANXA1 following DARTS assay treatment. *p < 0.05 vs. Control, ^#^ *p* < 0.05 vs. Pronase. (F) Western blot analysis of ANXA1 degradation at indicated temperatures. *p < 0.05 vs. DMSO. (G) qRT‐PCR analysis of ANXA1 mRNA expression. *p < 0.05 vs. csh‐NC. (H) Western blot analysis of ANXA1 protein expression. (I) Flow cytometry analysis of apoptosis rate. (J) Western blot analysis of ZO‐1 and Occludin protein expressions. (K) ELISA measurement of inflammatory factors (IL‐6, IL‐1β, and TNF‐α) in cell supernatants. Data are presented as mean ± SD (*n* = 3). *p < 0.05 vs. Control, ^#^ *p* < 0.05 vs. OSF, and *p* < 0.05 vs. pOSF+Kaempferol+sh‐N C.

We conducted molecular docking simulations to predict the binding interaction between kaempferol and ANXA1, with the optimal binding conformation shown in Figure 4A. Kaempferol stably bound to both human and rat ANXA1 with binding energies of −7.1 and−8.0 kcal/mol, respectively. Kaempferol formed stable interactions in the hydrophobic pocket of ANXA1, primarily mediated by van der Waals contacts and hydrogen bonding with neighboring residues. To establish an optimal concentration for the OSF in vitro model, human oral mucosal epithelial cells were treated with various concentrations of arecoline (0, 0.05, 0.10, 0.15, 0.20, and 0.30 mM) for 24 h. Arecoline treatment reduced epithelial cell viability in a concentration‐dependent manner (0–0.20 mM). However, 0.30 mM arecoline showed similar effects to 0.20 mM, so 0.20 mM was selected for subsequent experiments (Figure 4B). Arecoline also dose‐dependently suppressed ANXA1 protein expression in epithelial cells (Figure 4C). For therapeutic concentration screening, epithelial cells were treated with various concentrations of kaempferol (0, 1.25, 2.5, 5, 10, 20, and 40 μM) for 24 h. Concentrations up to 20 μM showed no significant effect on cell viability, while 40 μM significantly inhibited epithelial cell activity (Figure 4D). To verify direct binding between kaempferol and ANXA1, pronase was added to cell lysates incubated with kaempferol. Kaempferol was shown to protect ANXA1 from pronase digestion, thereby enhancing its protein stability (Figure 4E). CETSA assays further confirmed this interaction, showing that kaempferol stabilized ANXA1 at various temperatures in thermally denatured cell lysates (Figure 4F).

We then investigated ANXA1 knockdown in the in vitro OSF model. Among three targeted shRNA plasmid vectors tested, site #3 demonstrated the highest knockdown efficiency and was therefore chosen for further experiments (Figure 4G). As expected, ANXA1 knockdown reversed therapeutic effects of kaempferol, suppressing ANXA1 synthesis in arecoline‐treated epithelial cells (Figure 4H). Consistently, ANXA1 knockdown abolished anti‐apoptotic effects of kaempferol in arecoline‐treated cells (Figure 4I). Furthermore, kaempferol prevented arecoline‐induced downregulation of ZO‐1 and Occludin expressions, but this protection was reversed by ANXA1 knockdown (Figure 4J). ELISA analysis of inflammatory factors in cell supernatants revealed that kaempferol suppressed arecoline‐induced inflammatory cytokine release, an effect that was counteracted by ANXA1 knockdown (Figure 4K). In summary, kaempferol bound to ANXA1 to mitigate arecoline‐induced oral mucosal epithelial cell damage by reducing apoptosis and inflammation, with ANXA1 knockdown reversing these therapeutic effects.

### 3.5 Kaempferol Suppressed Fibroblast Activation via ANXA1/FPR2 Signaling to Restore Epithelial‐Fibroblast Homeostasis

**FIGURE 5:**
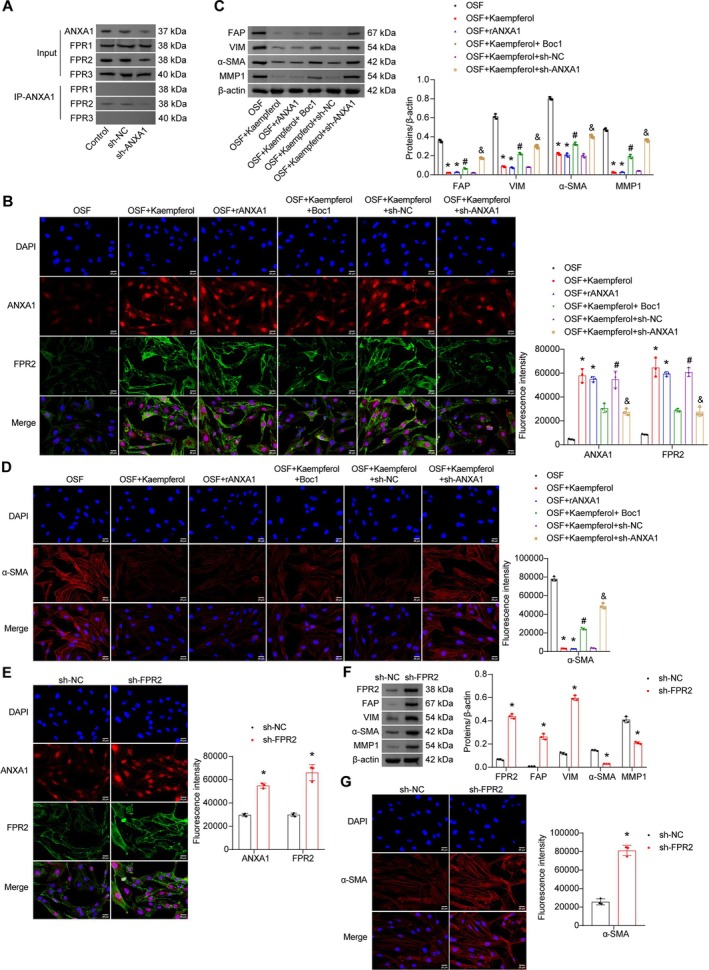
Kaempferol restored epithelial‐fibroblast homeostasis by suppressing fibroblast activation via ANXA1/FPR2 signaling upregulation. (A) Co‐IP analysis of specific binding between ANXA1 and FPR family members (FPR1, FPR2, and FPR3). Input: Lysates from fibroblasts co‐cultured with the indicated epithelial cell groups, probed for ANXA1, FPR1, FPR2, and FPR3. IP‐ANXA1: Immunoprecipitates from fibroblast lysates (co‐cultured with the indicated epithelial cell groups) using ANXA1 antibody, probed for FPR1, FPR2, and FPR3. (B) IF staining of ANXA1 and FPR2 expressions in fibroblasts (scale bar = 25 μm). (C) Western blot analysis of fibroblast activation markers (FAP, VIM, α‐SMA, and MMP1). (D) IF staining of α‐SMA expression in fibroblasts (scale bar = 25 μm). *p < 0.05 vs. OSF, ^#^ *p* < 0.05 vs. OSF + Kaempferol, and *p* < 0.05 vs. OSF + Kaempferol+sh‐NC. (E) IF staining of ANXA1 and FPR2 expressions in fibroblasts (scale bar = 25 μm). (F) Western blot analysis of fibroblast activation markers (FAP, VIM, α‐SMA, and MMP1). (G) IF staining of α‐SMA expression in fibroblasts (scale bar = 25 μm). Data are presented as mean ± SD (*n* = 3). *p < 0.05 vs. sh‐N C.

Research on esophageal lesions has demonstrated that ANXA1 controls fibroblast‐to‐myofibroblast transition through ligand‐receptor interactions with FPR2 (Chen et al. 2023b), prompting us to investigate potential ANXA1‐FPR interactions in human oral mucosal fibroblasts. It was worth noting that Co‐IP results showed that after co‐culturing with oral mucosal epithelial cells treated differently, ANXA1 in oral mucosal fibroblasts specifically bound to FPR2, while no significant interaction was detected with FPR1 or FPR3 receptors of the same family. This confirmed that FPR2 was the main functional receptor of ANXA1 in the context of OSF. Moreover, this specific binding was significantly diminished upon ANXA1 knockdown (Figure 5A). To examine epithelial‐fibroblast crosstalk, we co‐cultured untreated oral mucosal fibroblasts with differentially treated oral mucosal epithelial cells. IF staining further supported this ligand‐receptor interaction, showing membrane co‐localization of ANXA1 and FPR2 in oral mucosal fibroblasts. Both kaempferol and rANXA1 counteracted arecoline‐induced downregulation of ANXA1 and FPR2 expression. However, these effects were reversed by either ANXA1 knockdown or Boc1 treatment (a selective FPR2 antagonist) (Figure 5B). Subsequent analysis of fibroblast activation markers demonstrated that both kaempferol and rANXA1 suppressed fibroblast activation, as evidenced by downregulated expression of FAP, VIM, α‐SMA, and MMP1. These effects were similarly abolished by ANXA1 knockdown or Boc1 treatment (Figure 5C,D). We also investigated the effect of ANXA1‐FPR2 binding interruption on inhibition of kaempferol on fibroblast activation and found that when FPR2‐knockdown oral mucosal fibroblasts were co‐cultured with oral mucosal epithelial cells treated with arecoline and kaempferol, the co‐localization of ANXA1 and FPR2 on the fibroblast cell membrane decreased, accompanied by downregulation of the protein expressions of activation markers (FAP, VIM, α‐SMA, and MMP1) (Figure 5E–G). The above results indicated that kaempferol promoted the production of ANXA1 in epithelial cells, enhanced the specific binding of ANXA1 and FPR2 on fibroblasts, inhibited the activation of fibroblasts, and thereby maintained epithelial‐fibroblast homeostasis.

## 4 Discussion

OSF is a persistent inflammatory disorder characterized by progressive fibrosis and a significant potential for malignant progression. Currently, clinical symptoms and pathological examinations remain the primary means for diagnosing OSF. With the advancement of biotechnology, research on biomarkers of OSF has become increasingly active, which facilitates the diagnosis of both OSF and its malignant transformation (Y. W. Shen, Shih, et al. 2020). In this study, we demonstrated that JDK and its active ingredient, kaempferol, alleviated OSF by restoring epithelial‐fibroblast homeostasis and inhibiting neutrophil infiltration through the ANXA1/FPR2 axis. Our findings offered new insights into the molecular mechanisms underlying OSF progression and highlighted ANXA1 as a potential therapeutic target for the treatment of OSF.

Previous studies have shown that JDK and its active ingredients (such as quercetin) can prevent OSF and carcinogenesis by inhibiting signaling pathways such as AR/eIF5A2 or Notch1 (Dai et al. 2025; Zhou et al. 2023). In this study, we found that arecoline could induce oral epithelial atrophy, collagen deposition, and fibroblast activation, and promote the progression of OSF, which was consistent with the reports in the literature (S. Tang et al. 2024). The intervention of JDK and its component kaempferol significantly inhibited these changes and improved the mouth opening function of OSF rats, confirming its anti‐fibrotic efficacy. This was consistent with the reports of the anti‐fibrotic effect of kaempferol in liver, kidney, and lung diseases (Cao et al. 2023; Guan et al. 2023; Jiang et al. 2023; X. Zhang et al. 2024). In general, kaempferol is an excellent natural anti‐fibrotic agent.

In subsequent research, we further explored the key downstream effector molecules of kaempferol. After administering arecoline, we observed a reduced ANXA1 expression in both the oral mucosa and serum of rats, suggesting a potential association between ANXA1 expression and OSF. Over the past few years, emerging studies have demonstrated a close link between ANXA1 and fibrosis (Yan et al. 2022). Research has found that a downregulated state of ANXA1 expression is more likely to contribute to the development of OSCC (L. Zhang et al. 2009). Our study revealed that after downregulating ANXA1 expression, rats with OSF exhibited oral epithelial atrophy, collagen deposition, fibroblast activation, and severely impaired mouth opening function. Through further molecular docking, DARTS, and CETSA experiments, we confirmed that kaempferol directly bound to ANXA1 and regulated its protein expression. This interaction is crucial for improving the barrier integrity of oral mucosal epithelial cells and reducing epithelial cell apoptosis induced by arecoline. In a word, our results indicated that ANXA1 was a key downstream effector molecule of kaempferol. Targeted regulation of ANXA1 expression represented an effective therapeutic approach for OSF.

Inflammation is the core driving force of fibrosis, and inhibiting neutrophil recruitment is one of the strategies to alleviate fibrosis (Yan et al. 2022). ANXA1 is an endogenous anti‐inflammatory mediator that can inhibit neutrophil migration by interacting with the FPR2 receptor (D'Acquisto et al. 2008; Sugimoto et al. 2016; Walther et al. 2000). It is worth noting that the ANXA1/FPR2 signaling has been reported as an important communication mechanism between epithelial cells and fibroblasts (Chen et al. 2023a). In this study, arecoline induced fibroblast activation, significant neutrophil aggregation, and release of pro‐inflammatory cytokines in rat oral mucosa. JDK and its active ingredient kaempferol promoted the expression of ANXA1 in oral mucosal epithelial cells to inhibit neutrophil aggregation and release of pro‐inflammatory cytokines. Further binding of ANXA1 to FPR2 on fibroblasts through cell co‐culture inhibited their further activation into myofibroblasts. However, ANXA1 knockdown reversed the therapeutic effect of JDK and its active ingredient kaempferol on OSF. These results indicated that kaempferol, a component of JDK, could restore epithelial fibroblast homeostasis and inhibit neutrophil recruitment to improve OSF by specifically regulating the ANXA1/FPR2 axis. This pivotal insight is the core innovation point of this study.

The findings of this study exhibit promising translational potential. As a natural flavonoid widely present in foods, the safety profile of the active ingredient kaempferol provides a foundation for the possible development of dietary supplements for OSF. The study further demonstrates that kaempferol acts by upregulating the endogenous pro‐resolving mediator ANXA1 and activating its receptor FPR2. This strategy of utilizing dietary components to enhance the body's own inflammation‐resolving pathways aligns with a host‐directed, low‐toxicity therapeutic paradigm, which may offer greater safety and specificity compared to broad‐spectrum immunosuppression. Future research could explore the development of agonists targeting this pathway or the optimization of kaempferol formulations to facilitate its translation into clinical applications.

Although this study revealed the potential therapeutic mechanism of JDK and its active ingredient kaempferol in OSF through the regulation of ANXA1, there are still the following limitations. First, this study was primarily conducted using an arecoline‐induced OSF rat model and in vitro cell models. While this model can simulate some pathological features of OSF, it still differs from the complex etiology and pathogenesis of human OSF, which might limit the extrapolation of the experimental results. Future research should further validate the findings in human OSF tissue samples. Second, this study confirmed that kaempferol inhibited fibroblast activation by upregulating the ANXA1/FPR2 signaling pathway, but the investigation into other potentially involved molecules within this pathway and their interaction networks remains insufficient. For example, the specific downstream genes and proteins regulated by the ANXA1/FPR2 signaling pathway, as well as potential cross‐regulations with other signaling pathways, require further exploration. Finally, this study focused on basic experimental research and, while providing new insights and potential targets for OSF treatment, did not address the safety and efficacy of JDK and kaempferol in clinical applications. Aspects such as drug dosage, administration methods, long‐term side effects, and the combined effects with other treatment approaches remain to be evaluated. Subsequent large‐scale clinical trials are needed to facilitate their clinical translation.

## 5 Conclusion

In summary, JDK and kaempferol ameliorated arecoline‐induced OSF by upregulating ANXA1, which suppressed neutrophil recruitment and fibroblast activation via the FPR2 pathway. These findings highlight ANXA1 as a therapeutic target and support the potential of traditional Chinese medicine in OSF treatment.

## Author Contributions

**Yanli Liu:** data curation. **Zhaoyong Hu:** data curation, validation, investigation, writing – review and editing. **Jin Tan:** data curation, conceptualization, methodology, funding acquisition, writing – review and editing, project administration, supervision, resources. **Tao Zhou:** writing – review and editing. **Yao Xiao:** methodology, validation, visualization, writing – review and editing, investigation, writing – original draft, data curation, formal analysis. **Yisi Tan:** data curation, validation, writing – review and editing. **Ruiyi Chen:** data curation.

## Funding

This work was supported by a grant from the National Natural Science Foundation of China (No. 82374530) and the Outstanding Youth Scientific Research Project of Hunan Provincial Department of Education (No. 25B0359).

## Ethics Statement

No clinical sample was used in this study. All experimental procedures involving animals followed the ARRIVE guidelines and the Laboratory Animal Ethics Committee of Hunan University of Chinese Medicine approved this study (No. HN‐LL‐GZR‐2023‐43).

## Consent

The authors have nothing to report.

## Conflicts of Interest

The authors declare no conflicts of interest.

## Data Availability

The data that support the findings of this study are available from the corresponding author upon reasonable request.
